# First person – Tabea Soelter and Emma Jones

**DOI:** 10.1242/dmm.052868

**Published:** 2026-02-27

## Abstract

First Person is a series of interviews with the first authors of a selection of papers published in Disease Models & Mechanisms, helping researchers promote themselves alongside their papers. Tabea Soelter and Emma Jones are co-first authors on ‘ Cell-type-specific alternative splicing in the brain and kidney of a *Setbp1*^S858R^ Schinzel–Giedion syndrome mouse’, published in DMM. Tabea is a computational biologist in the lab of Brittany Lasseigne at The University of Alabama at Birmingham, Birmingham, AL, USA, using transcriptomics and bioinformatics to uncover the mechanisms underlying age-associated diseases, specifically those with a neurodegenerative phenotype. Emma conducted the research described in this article while a PhD student in Brittany Lasseigne's lab at The University of Alabama at Birmingham. She is now a postdoc in the lab of Joseph Dougherty at Washington University in St Louis, St Louis, MO, USA, using transcriptomics to understand the mechanisms underlying neurodevelopmental and psychiatric disorders.


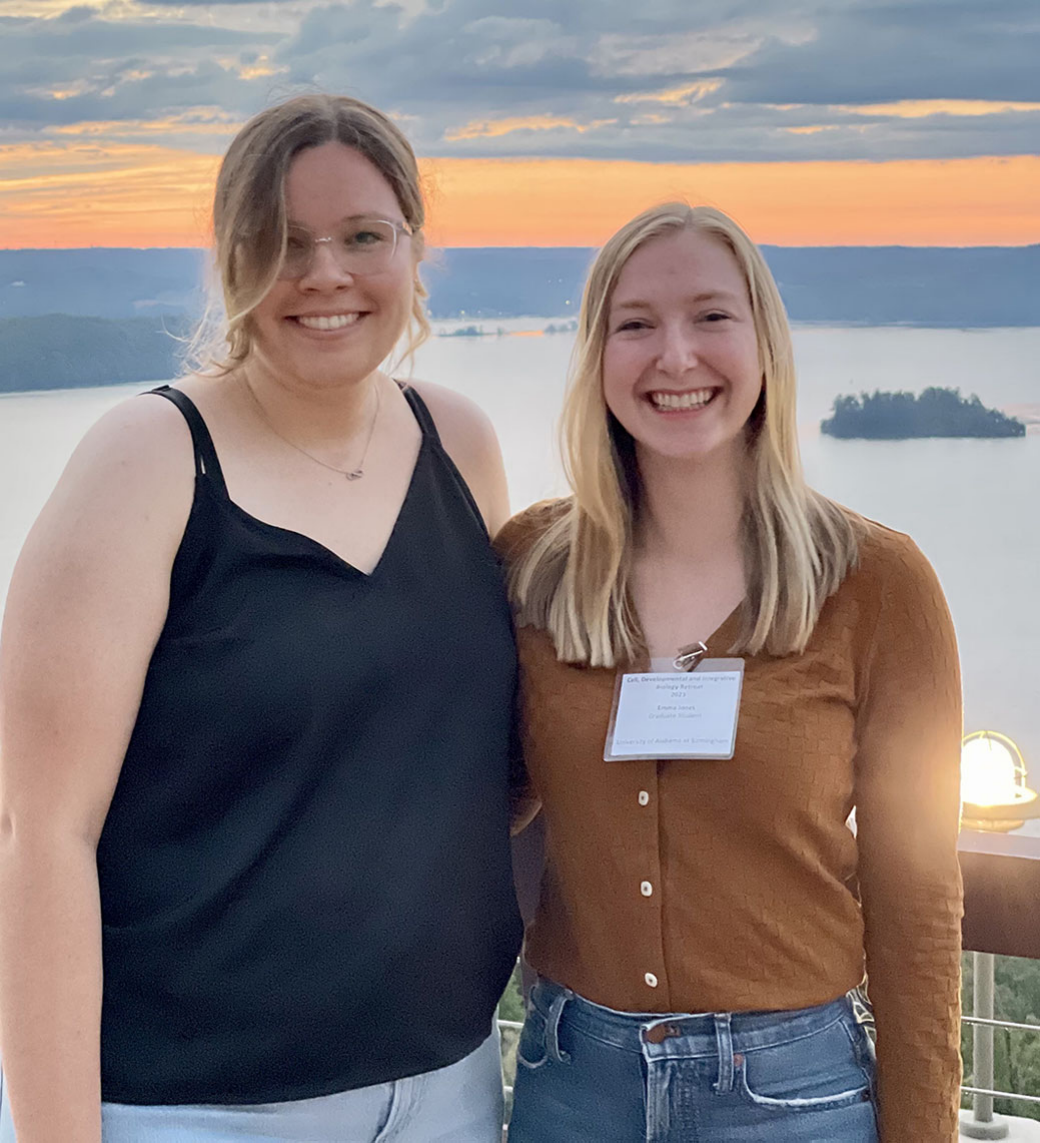


**Tabea Soelter (left) and Emma Jones (right)**

**Who or what inspired you to become a scientist?**

**E.J.:** I was interested in how mental health disorders arise and why patients often have to try multiple different psychiatric medications. I wanted to know how the brain worked, which sparked my interest in psychology, then in neurotransmitters and molecules, and eventually led me to molecular genetics. Now, I spend a lot of time thinking about how we can better understand the brain to develop treatments and medications that work for everyone.

**T.S.:** Growing up, my grandfather was diagnosed with Parkinson's disease, which sparked my initial interest. Then, in high school, I attended an interactive guest lecture on the brain from a scientist at a local university. Her lecture, combined with my feelings of helplessness in my grandfather's Parkinson's diagnosis as a child, motivated my decision to pursue a career in science.

**What is the main question or challenge in disease biology you are addressing in this paper? How did you go about investigating your question or challenge?**

**T.S./E.J.:** Due to SETBP1's regulation of splicing factors and its neurodevelopmental role, we sought to investigate whether the Schinzel–Giedion syndrome (SGS) patient variant can also impact alternative splicing. In previous work from our lab, we identified many cell-type-specific perturbations in gene expression and regulation, so we hypothesized that these perturbations would also affect alternative splicing.

**How would you explain the main findings of your paper to non-scientific family and friends?**

**T.S./E.J.:** We used existing RNA-sequencing data and carried out computational analyses to figure out whether RNA processes are different in a rare neurological disorder using mice. We confirmed that genes that get turned on and off actually do so to different degrees, like a dimmer rather than a switch in SGS, an ultra-rare disorder affecting young children.

... genes that get turned on and off actually do so to different degrees, like a dimmer rather than a switch in [Schinzel–Giedion syndrome] ...

**What are the potential implications of these results for disease biology and the possible impact on patients?**

**T.S./E.J.:** Unfortunately, there is currently no treatment or cure for SGS, suggesting that investigating novel molecular mechanisms, such as alternative splicing, could lead to a better understanding of the disease and, ideally, new treatment options in the future. Since our study is the first to evaluate cell-type-specific alternative splicing patterns in a mouse model of SGS, we expect our results will generate interest in further investigating alternative splicing in SGS to pinpoint how it might lead to or affect this neurodevelopmental disorder. We used a single mouse model and short-read single-nuclei RNA sequencing, so we hope that our early findings will encourage others to investigate alternative splicing using additional models, human data (if possible) and long-read RNA-sequencing, which can measure full-length transcripts and yield more accurate findings. We are especially hopeful that our web application, which includes all our findings, will facilitate such future studies.

**Figure:**
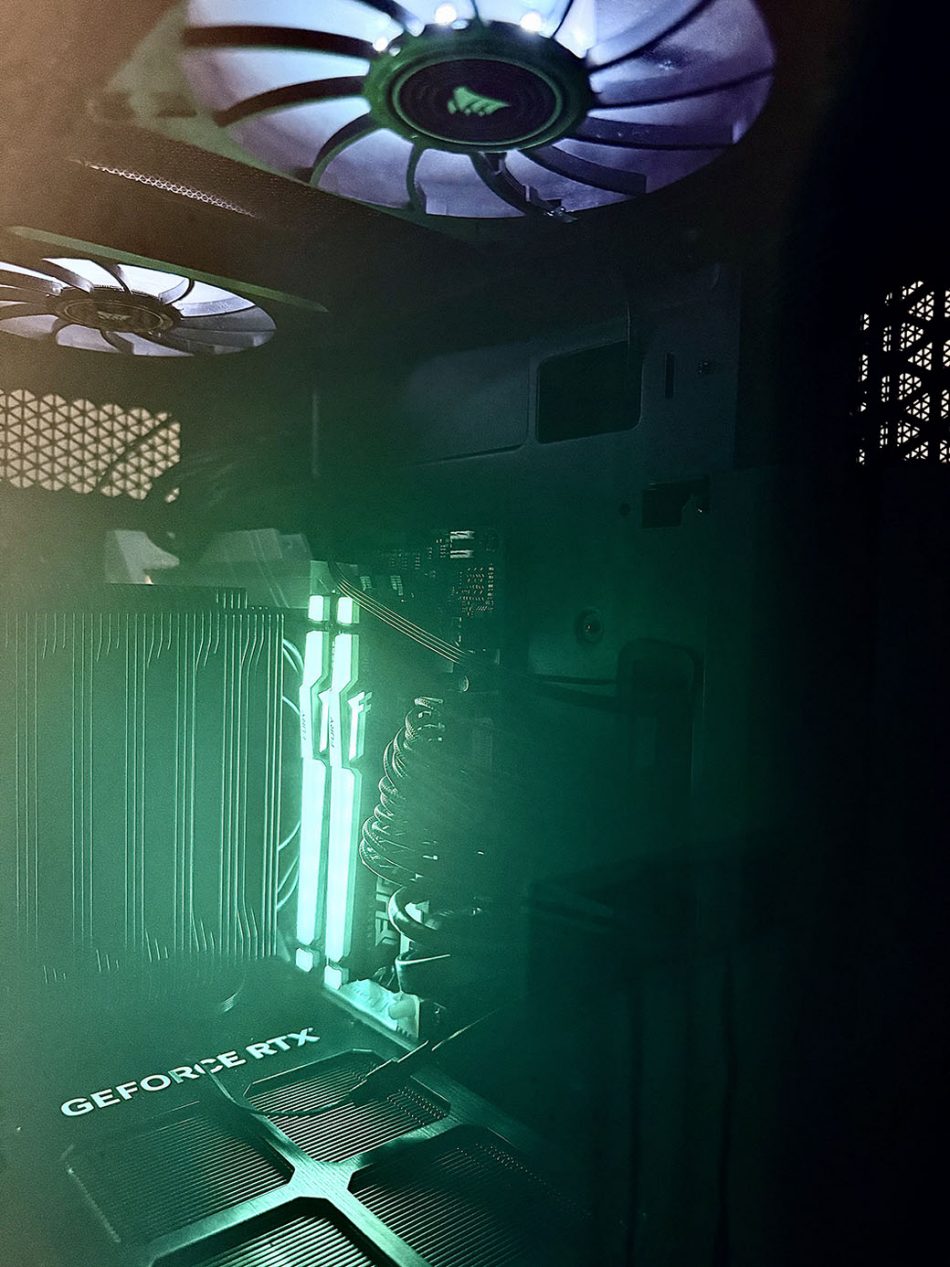
High-performance computing hardware powering Oxford Nanopore Technologies' long-read RNA-sequencing basecalling and subsequent alternative splicing analyses in *SETBP1*-related disorders.

**Why did you choose DMM for your paper?**

**T.S./E.J.:** DMM publishes interdisciplinary research across many diseases and cutting-edge resources. Additionally, DMM featured a special issue titled ‘Translating Multiscale Research in Rare Disease’ in June 2024. Since our research focuses on SGS, an ultra-rare disease, and we include an interactive web app of our results, we believe that DMM's interdisciplinary approach and its previous commitment to publishing rare disease research are a good fit for our findings.

**Given your current role, what challenges do you face and what changes could improve the professional lives of other scientists in this role?**

**E.J.:** The current instability of federal funding is a major problem for many scientists. The lack of new jobs at research institutes and universities means that fewer student, postdoc, staff and faculty positions are opening up, and there is less room for professional growth. In addition, National Institutes of Health funding rates are so low that high-quality grants can still get rejected, because there are more good ideas than available funds. Increasing public trust and support of science could help stabilize federal funding and keep scientists like us employed.

**T.S.:** Research scientists often work long hours and face intense workloads and funding pressures. This constant strain from multiple angles leads to high rates of burnout, depression and anxiety. I think that if research institutions prioritized preventative measures that mitigate burnout among their workforce, this could greatly improve their professional lives, as scientists operating at optimal wellbeing consistently demonstrate enhanced productivity and produce higher-quality research outcomes.

… high-quality grants can still get rejected, because there are more good ideas than available funds

**What's next for you?**

**E.J.:** I plan to analyse 100+ single-cell and spatial transcriptomic mouse datasets at the Washington University Scalable Mouse Assay Center, which is part of the National institute of Mental Health-funded Scalable and Systematic Neurobiology of Psychiatric and Neurodevelopmental Disorder Risk Genes (SSPsyGene) consortium. SSPsyGene aims to study 250+ neurodevelopmental and psychiatric risk genes (many of which cause syndromic neurodevelopmental disorders) across multiple data modalities, including transcriptomics, electrophysiology, whole-brain imaging, cell morphology and behaviour. Eventually, I would like to become an independent investigator studying complex, polygenic neurodevelopmental and psychiatric disorders using these kinds of data-driven approaches.

**T.S.:** In line with my career goals, I recently transitioned into a staff position as a computational biologist. In this position, I am excited to continue researching and, hopefully, expand our understanding of the underlying mechanisms that drive age-associated and neurodegenerative diseases through computational and genomic approaches.

**Tell us something interesting about yourself that wouldn't be on your CV**

**E.J.:** I help manage a Minecraft server that has been running for over 10 years.

**T.S.:** I am an avid fantasy reader.
